# Anti-fibrotic effect of *Sedum sarmentosum* Bunge extract in kidneys via the hedgehog signaling pathway

**DOI:** 10.3892/mmr.2022.12658

**Published:** 2022-02-25

**Authors:** Yongheng Bai, Cunzao Wu, Weilong Hong, Xing Zhang, Leping Liu, Bicheng Chen

Mol Med Rep 16: 737–745, 2017; DOI: 10.3892/mmr.2017.6628

Subsequently to the publication of the above paper, an interested reader drew to the authors’ attention that certain of the data featured in Fig. 1A on p. 740 had already appeared in another publication written by the same authors [*Sedum sarmentosum* Bunge extract exerts renal anti-fibrotic effects *in vivo* and *in vitro.* Bai Y, Lu H, Wu C, Lin C, Liang Y and Chen B. Life Sci: 105, 22–30, 2014]. The authors have been able to re-examine their original data, and realized that certain of the data were misplaced in Fig. 1 in the above paper on account of mishandling their data.

The revised version of Fig. 1 in shown on the next page, featuring the corrected data in Fig. 1A for the HE staining SSBE- and Vehicle-UUO experiments, and the Masson staining/SSBE and Vehicle/Sham and UUO experiments (all four data panels), the TGF-β1 experiments in Fig. 1C (all four data panels) and the four data panels in Fig. 1D. Note that the errors made during the assembly of this figure did not adversely affect the overall conclusions reported in the study. The authors are grateful to the Editor of *Molecular Medicine Reports* for allowing them the opportunity to publish this corrigendum, and all authors agree to the publication of this corrigendum. Furthermore, they wish to apologize to the readership of the Journal for any inconvenience caused.

## Figures and Tables

**Figure 1.**
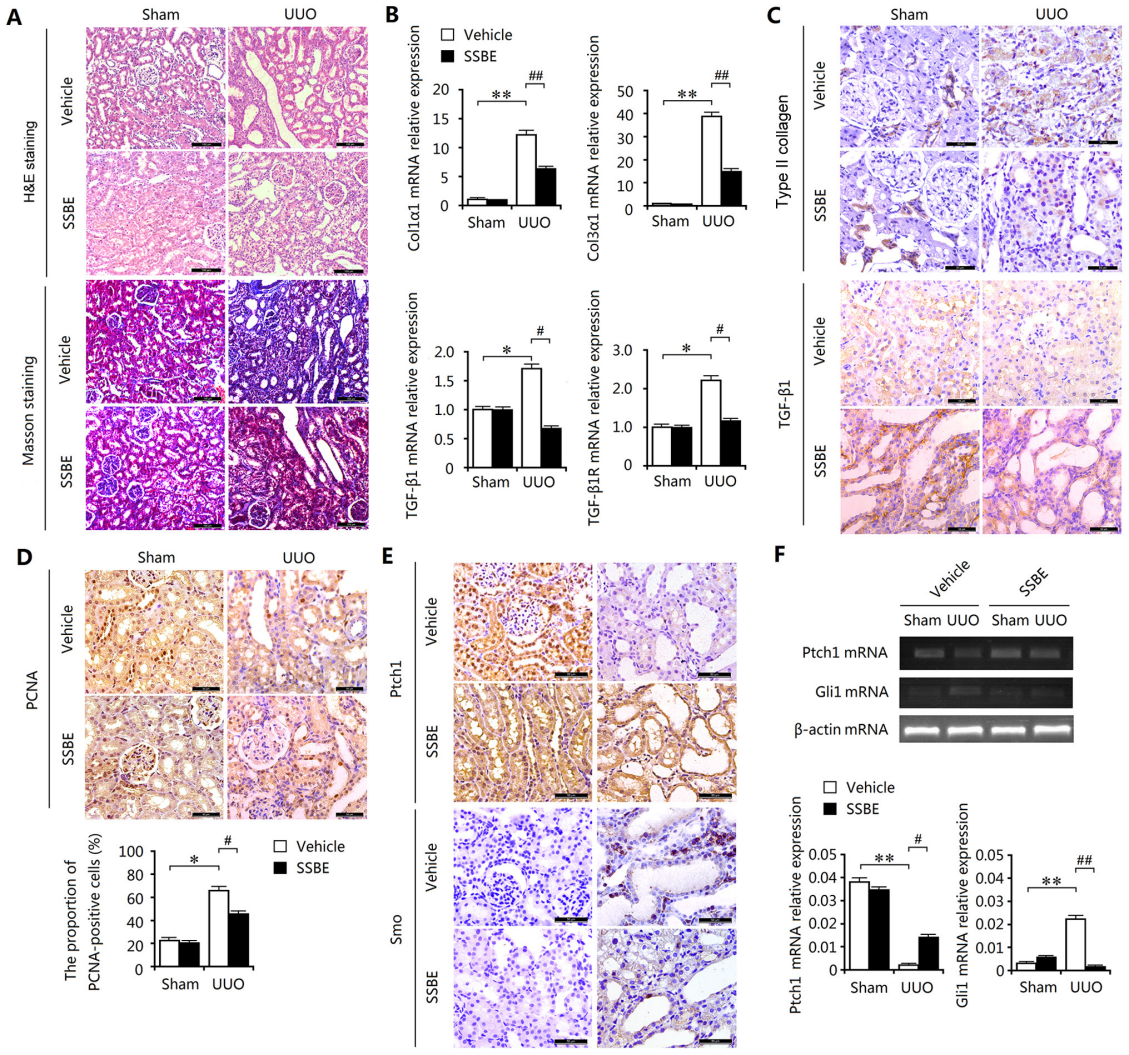
SSBE inhibits hedgehog signaling activity and alleviates interstitial fibrosis in UUO kidneys. (A) H&E and Masson trichrome staining indicated marked kidney injury and excessive accumulation of total collagen in UUO kidneys, but SSBE administration alleviated this effect. Scale bar, 100 µm. (B) Enhanced mRNA expression levels of Col1α1, Col3α1, TGF-β1 and TGF-β1R in UUO kidneys, determined by reverse transcription quantitative polymerase chain reaction, were inhibited by SSBE treatment. (C) Immunochemical staining indicated upregulated expression of Col1α1 and TGF-β1 in UUO kidneys, which were alleviated following SSBE administration. Scale bar, 50 µm. (D) SSBE decreased PCNA expression in kidney tissues of UUO rats. Scale bar, 50 µm. (E) SSBE administration inhibited UUO-induced downregulated protein expression levels of Ptch1, and upregulated expression of Smo. Scale bar, 50 µm. (F) UUO decreased mRNA expression levels of Ptch1 and increased expression of Smo, but were inhibited by SSBE treatment. Data are presented as the mean ± standard error. *P<0.05, **P<0.01 vs. sham; ^#^P<0.05, ^##^P<0.01 vs. vehicle. H&E, hematoxylin and eosin; UUO, unilateral ureteral obstruction; Col1α1, type I collagen; Col3α1, type III collagen; TGF-β1, transforming growth factor-β1; TGF-β1R, transforming growth factor β1 receptor; SSBE, *Sedum sarmentosum* Bunge; PCNA, proliferating cell nuclear antigen; Ptch1, protein patched homolog 1; Smo, smoothened.

